# A qualitative exploration into the experience of mindfulness in moderate-severe persistent depression

**DOI:** 10.1371/journal.pone.0323294

**Published:** 2025-06-09

**Authors:** Timothy Sweeney, Elena Nixon, Richard Morriss, Patrick Callaghan

**Affiliations:** 1 Adult Mental Health Directorate, Nottinghamshire Healthcare Foundation Trust, Nottingham, United Kingdom; 2 Mental Health and Clinical Neurosciences, Institute of Mental Health, School of Medicine, University of Nottingham, Nottingham, United Kingdom; 3 School of Applied Sciences, London South Bank University, London, United Kingdom; Keele University, UNITED KINGDOM OF GREAT BRITAIN AND NORTHERN IRELAND

## Abstract

Depression is a common and growing mental health problem, with around 5% of the world’s population experiencing an episode of depression during their lifetime. Relapse rates are high, with around half experiencing more than one depressive episode and a further 10–20% experiencing a chronic and persistent depression. Mindfulness has been incorporated into treatments for depression and several studies have explored the impact of mindfulness training on depressive symptomatology and recurrence. However, to date no studies have looked at the changing relationship between mindfulness and depression in those naïve to mindfulness training. 20 participants with moderate-to-severe persistent depression were interviewed to explore their experience of mindfulness in the context of low mood. Thematic analysis captured six themes highlighting changes in mindfulness relating to the onset of depression. Themes included: behavioural withdrawal; perceptual detachment from one’s experience; intentional reduction in awareness; increased self-criticism; mind racing; impaired cognitive performance. Thematic analysis suggested that mindfulness reduces in the context of moderate-to-severe persistent depression. This appears to occur indirectly as the consequence of depression-related processes, e.g., rumination and experiential avoidance, but also arises as a deliberately instigated self-protective strategy. However, findings seemed to indicate that reduced mindfulness maintains and intensifies depressive experience. Despite growing evidence of the value of mindfulness approaches for those with more chronic and severe depression, study findings suggest that introducing mindfulness to this population may be particularly challenging due to the intensity of symptomatology potentially obstructing access to a mindful perspective. Findings bear important implications for the treatment of depression and can inform future intervention development and delivery.

## Introduction

Depression is the most common mental health problem worldwide [1]. At any one point, around 5% of the world’s population will be experiencing an episode of depression [2]. Despite the evidence showing that individuals with depression may remit either spontaneously or following healthcare interventions, a significant proportion fail to achieve remission [3]. Incomplete recovery is common with up to half of the affected individuals still meeting criteria for diagnosis one year after onset due to high relapse rates [4], with some experiencing a pattern of depression characterised by recurrent and frequent episodes, occurring with no clear trigger. Up to 20% of individuals with depression will experience a chronic and persistent depression lasting several years [5].

Defined as the capacity to pay deliberate attention to present moment experience without judgment [6], mindfulness has been incorporated into psychotherapeutic approaches evidenced to be effective for the treatment of depression. Most notably mindfulness meditation practices have been combined with cognitive therapy exercises in an eight-week group programme: Mindfulness-based Cognitive Therapy (MBCT) [7]. This course was specifically designed to reduce episodes of depression in vulnerable individuals. MBCT involves attentional training, using mindfulness meditation. Through this teaching, participants with a history of depression become more aware of momentary changes in affect, allowing them to intervene at the point at which low mood typically escalates into depressive relapse (*ibid*). Early Randomised Controlled Trials (RCTs) on MBCT efficacy consistently showed significant reductions in the risk of relapse for those with a history of three or more depressive episodes compared to Treatment-As-Usual (TAU) [e.g., 7–10]. In 2016, a meta-analysis (*n = *1,258) concluded that MBCT is an effective intervention for relapse prevention in recurrent depression in remission, compared to TAU and compared to other active treatments [11]. In addition to investigating the impact of MBCT on relapse prevention, several trials have also tested the effects of MBCT on those in a current episode of depression, demonstrating significant reductions in acute depressive symptomatology [12–18]. Group-based mindfulness meditation courses, such as MBCT, are now recommended in UK national health guidelines for individuals with mild to moderate and recurring depression in remission [5]. Trials of MBCT for chronic depression [18] and TRD [19] provide a growing evidence base of the value of mindfulness training for those with more persistent and severe forms of depression.

In addition to quantitative trials, a number of qualitative studies have explored the impact of mindfulness training offered through MBCT programmes on people with depressive disorders. Within these, the attitude acceptance is frequently explored, with participants reporting a less judgemental attitude to themselves post intervention [20–28]. Greater awareness of thoughts, feelings, and bodily sensations are further reported [20; 21; 23; 29], facilitating identification of early warning signs of low mood and enabling depressive relapse-preventing actions to be taken [23]. Participants on such programmes also describe the development of an increasingly decentred perspective, allowing oneself to dissociate from thoughts and feelings, and an associated reduction in rumination [29]. The development of heightened self-awareness, attitudes and skills attained through mindfulness training are additionally reported in studies to provide a greater sense of control, empowerment and agency as participants feel increasingly able to influence their mood directly, rather than being submissive to processes beyond their control [23; 25; 26; 27; 30]. However, while studies so far have explored participants’ experience of engaging in MBCT for depression, to the best of the authors’ knowledge, there are no qualitative studies exploring the relationship between mindfulness and depression in those naïve to mindfulness training. Such an exploration may provide greater understanding of naturally occurring alterations in levels of mindful awareness in the context of worsening mood, and increase our understanding of the role, and potential value, of mindfulness in the experience of depression. Such understanding may provide the basis for improvements in the management of depression, for example by optimising timing of mindfulness-based interventions and suggesting potential adaptations to these. The aim of this study was therefore to explore in depth participants’ experience of mindfulness during depressive episodes and their perceived impact of depressive symptoms on their levels of mindfulness.

## Materials and methods

### Participants and recruitment procedure

The study used a qualitative approach embedded within a large scale, multi-centre randomised controlled trial (RCT: *n = *187) which was part of a government initiative aiming to improve integration of research findings into clinical practice. This trial was an investigation into the impact of combined expert pharmacological prescribing and CBT for people with severe and persistent depression and is registered at ClinicalTrials.gov (NCT01047124) and the ISRCTN registry (ISRCTN10963342). A subsample of participants who took part in the trial (*n = *20) was recruited for this part of the study. Inclusion criteria for the RCT were: meeting criteria for at least moderate depression (five out of nine symptoms of depression: [31]; a minimum Hamilton Depression Rating Scale score of 16 indicating at least moderately severe depression HDRS [32]; a score of 60 or less on the Global Assessment of Functioning Scale (GAF [33]) indicative of significant social and occupational impairment; good working knowledge of English; an ability and willingness to provide oral and written informed consent to participation in the study; aged 18 years or older. Exclusion criteria were: receiving emergency care for suicide risk, risk of severe neglect or homicide risk; patients were not excluded because of such risk provided the risk was adequately contained within their current care setting and the primary medical responsibility for care remained with the referring team; unable to speak fluent English; being pregnant; experiencing unipolar depression secondary to a primary psychiatric or medical disorder. Additional criteria for the current qualitative study included the exclusion of participants who had been through mindfulness training. Mindfulness training involves cultivating greater levels of mindful awareness so that depression related phenomena can be more effectively managed. Such training might therefore interfere with participants experience and reporting of spontaneously occurring alterations in mindfulness in the context of deteriorating mood.

Participants in the RCT were receiving treatment in secondary mental health services from community mental health teams, outpatient, and in-patient units in three UK mental health trusts (hospitals). The participants’ healthcare professional considered them to be experiencing a primary unipolar depressive disorder for which they had received treatment in the preceding six months but remained depressed despite this. For the current study (*n = *20) purposive rather than convenience sampling was employed to ensure a sub-sample of individuals who were naive to mindfulness training were recruited in order to determine whether this concept is spontaneously volunteered as a feature of depressive experience. Potential participants were contacted by telephone by a member of the research team and were asked about their willingness to participate in semi-structured interviews exploring their experience of depression. They were additionally asked about any previous involvement with mindfulness training. This included asking about attendance on formal mindfulness courses, and any prolonged use of mindfulness self-help materials. An initial verbal explanation of the study was followed by provision of information sheets to assist participants in deciding whether or not to engage in the study. Following this (a period of at least 24 hours) their willingness to participate was determined via a telephone call. All participants approached were willing to participate in the study, and a meeting was subsequently arranged to complete the consenting process before proceeding with the interview procedure. All persons gave their informed written consent prior to their inclusion in the study. Recruitment and data collection began 10/09/2012 ending 17/03/2013. Prior to recruitment, a maximum number of 25 participants was deemed to be appropriate for the exploration of the data on this topic – subject to data saturation, as per previous protocol recommendations [34]. Ethics approval was obtained from the Health Research Authority (HRA) National Research Ethics Service (NRES), east midlands, Derby, UK and the University of Nottingham Faculty of Medicine and Health Sciences Research Ethics Committee. This study has therefore been performed in accordance with the ethical standards laid down in the 1964 Declaration of Helsinki and its later amendments.

### Data collection

Semi-structured interviews were conducted face to face with all participants, in a private place, i.e., a clinical office, the participant’s home or an alternative private location of the participants’ preference. Interviews were conducted by the author (TS), a highly trained mental health professional experienced in working with patients with severe levels of depression and distress. Interviews were recorded via a digital audio recorder, and were transcribed immediately after the interviews took place. Transcripts were anonymised, removing any sensitive or identifiable information that may have been inadvertently elicited during the interview; and were saved on a restricted-access, password-protected electronic folder, separately from participants’ demographic and consent data. All research and data management procedures complied with the ethical and research conduct guidelines under which the study protocol was approved.

Interviews began with a statement outlining the purpose of the interview. A semi-structured interview guide was used (see S1 File ‘Interview Schedule’ for details) which allowed flexibility to use prompting questions when appropriate, in keeping with the goals of thematic exploration [35]. Initial questions were intended to allow participants to fully delve into their experiences of depression. Subsequent interview questions were designed to identify whether facets of mindfulness were reported as an element of depressive experience. Initial interview questions were open and general, enquiring into the subjective experience of participants’ depression in order to explore whether aspects of mindfulness were volunteered spontaneously as a relevant component of this experience. Follow-up questions relating to mindfulness were then asked, referring to qualities embodied within facets of the Five Facets Mindfulness Questionnaire (FFMQ [36]), rather than asking directly about mindfulness. This approach ensured questions were not leading or closed and was intended to gather information about this topic in the participants’ words [35]. Of all existing mindfulness questionnaires, the FFMQ is arguably the most comprehensive due to its inclusion of items from several other mindfulness questionnaires and composition of five facets comprising an overarching construct of mindfulness. Facets consist of: (a) “Observe” (defined in terms of noticing internal and external experience); (b) “Describe” (the ability to articulate one’s experience); (c) “Nonjudge” (defined in terms of accepting thoughts and feelings without judgment or self-criticism); (d) “Nonreact” (the ability to be aware of thoughts and feelings without getting caught up in them; (e) “Actaware” (defined in terms of the ability to be aware of present moment experience, rather than being on autopilot). Example questions included:

*“Does depression affect the way you react to things? In what way?” (Nonreact).*

*“Please describe how depression affects the way you feel about yourself” (Nonjudge)*

*“What, if any, impact does depression have on the way you notice things?” (Observe)*

*“What, if any, impact does depression have on the way you pay attention to your experiences?” (Actaware)*

*“How, if at all, does depression affect your ability to function intellectually?” (Describe)*

### Data analysis

Transcribed data were rich in nature and were deemed appropriate for thematic analysis (TA) [34]. The six stages of thematic analysis, as outlined in the Braun & Clarke protocol guidelines *(ibid)* were followed. The process involves a data-driven approach to thematic analysis, where data are examined for codes and patterns and themes are allowed to emerge from the data [37]. First, data was transcribed by the author (TS), and transcripts were read and re-read with extracts identified relating to mindfulness, leading to the generation of initial codes. Several transcripts were also reviewed by an experienced qualitative researcher and co-author (EN), to increase accuracy and consistency of initial coding through an additional perspective [38]. Codes were then collated and relationships among codes were explored in order to lead to the generation of relevant themes. These were subsequently reviewed for face validity together with experienced healthcare professionals in this area and co-authors (RM and PC). Relationships between codes were explored and themes and related sub-themes were subsequently generated. Clear definitions and labels for each theme -and sub-theme where applicable- were put together and the thematic codebook was developed to denote how each theme and its sub-themes were derived from the generated codes (and subcodes) drawing upon illustrative extracts from participants’ responses (see S2 File ‘Example Codebook’ for further details). Following recruitment and analysis of 20 participant transcripts it was independently confirmed by the four researchers that no new themes were emerging, and that data saturation had been reached. Data collection consequently ceased at this point.

## Results

### Participant characteristics

After 20 participants had been recruited and interviewed, recruitment stopped as no new topics were being raised, indicating that data saturation had been reached. All participants were experiencing depression of at least moderate severity as measured by the HAMD [39], with a mean of 21.9 (5.45). Other participant descriptive characteristics are presented in Table 1. In response to questions about the onset and history of depressive disorder, some participants described a course of depression marked by repeated episodes (>3) and only rarely experiencing a full remission of symptoms between these. Others reported their depression had been constant since first onset, and they had been unable to continue working due to depression. Participants’ presentation was therefore characterised by substantial levels of persistent depression.

**Table 1:** Participant characteristics.

	Sample (*n* = 20)
Gender, Women: *n* (%)	7 (35%)
Age (in years): *M* (SD) Range 30–66	50.6 (11.02)
Severity of depression (HAMD): *M* (SD) Range 16–40	21.9 (5.45)
Duration of depressive disorder (in years): *M* (SD) Range 3–45 years	20.5 (11.21)
Mindfulness (FFMQ): *M* (SD) Range 89–118	97.4 (17.01)

### Themes and Sub-themes

Participants’ accounts of mindfulness whilst experiencing depression revealed six deductive themes: ‘Behavioural Withdrawal’, ‘Perceptual Detachment from One’s Experience’, ‘Intentional Reduction in Awareness’, ‘Increased Self-criticism’, ‘Mind Racing’ and ‘Impaired Cognitive Performance’. See Table 2 below for a list of themes and sub-themes.

**Table 2:** Themes and sub-themes.

Theme	Sub-theme
**1. Behavioural withdrawal**	1.1: Becoming disengaged from the world (global withdrawal)
	1.2: Avoiding contact with others for protection (social withdrawal)
	1.3: Retreating into one’s internal world (withdrawal into oneself)
**2. Perceptual detachment from one’s experience**	2:1 Not being aware of one’s internal experiences
	2.2: Shutting off from external experiences
**3. Intentional reduction in awareness**	3.1: Switching off awareness to insulate oneself from painful feelings
	3.2: Finding safety within one’s internal ‘painful’ state to avoid exposure to the external world
**4. Increased self-criticism**	4.1: A profound sense of worthlessness as a human being
	4.2: Self-blame for the presence of depressive symptoms
	4.3: Self-dislike of one’s physical appearance
**5. Mind racing**	5.1: Ruminating, Worrying and Relentless Thinking
	5.2: Preoccupation with thoughts preventing one from being in the present
**6. Impaired cognitive performance**	6.1: Losing the ability to find words to express oneself
	6.2: Losing the ability to think
	6.3: Reduced concentration on task at hand

### Theme 1: Behavioural withdrawal

The theme Behavioural Withdrawal relates participants’ accounts of disengaging from the external world in response to the onset of a depressive episode. This is vividly described by participants seeking to protect themselves from phenomena likely to trigger negative thoughts and feelings. Behavioural withdrawal can therefore be employed as a deliberate strategy to avoid further deterioration in mood and is described by participants as a central experience of depression. Participant comments highlighted three particular forms of behavioural withdrawal: actions to completely avoid contact with anything external (global withdrawal), a particular intention avoid contact with other people (social withdrawal), and absorption in ones’ internal world (withdrawal into oneself).

#### Sub-theme 1.1: Becoming disengaged from the world (global withdrawal).

When asked about changes occurring with the onset of depression many participants highlighted their tendency to try to completely avoid contact with external environments. Comments captured a desire to shut out the outside world as much as possible: *“You shut yourself away”* (P4, male, 52 years old).

Participants spoke of avoiding contact with the outside world by retreating into their bedroom as a frequent response to a drop in mood: *“I just get um, I just retreat to the bedroom, I draw the curtains, I pull the duvet over me and that’s that”* (P8, female, 43 years old).

Increased sleep is recognised as a symptom of depression in the DSM5 diagnostic criteria (30). In this study it was described as a specific form of withdrawing to avoid contact with unwanted phenomena including negative thoughts and feelings. This strategic use of sleep in response to depression is an example of participants’ intention to completely withdraw contact from anything that may exacerbate low mood. This is elucidated in the following quotes: *“One of the benefits of staying indoors and going to sleep is that it can cut out the risk of things spiralling downwards”* (P17, male, 57 years old).

*“Sleep is a good way of escaping everything”* (P18, male, 51 years old).

#### Sub-theme 1.2: Avoiding contact with others for protection (social withdrawal).

Comments about withdrawal are in response to questions focusing on how time is spent when depressed compared to when mood is improved. In addition to global withdrawal as identified above, participants also reported specifically avoiding social encounters when depressed, as described by one participant in response to the question *“Could you tell me about the experience of depression, what it consists of, the actual experience of it, as if you were describing it to a friend…”*

*“………….I wasn’t seeing anyone. I’d lock myself away from everyone I knew. I stopped seeing all my friends and family………….”* (P7, female, 30 years old).

This sub-theme represents a distinct form of withdrawal, focusing specifically on the drive to avoid contact with others: *“When I am unhappy I don’t want to be around people, so I just refuse to see anyone”* (P9, male, 62 years old).

Social withdrawal is a recognised feature of depression (17) and it is therefore unsurprising that this theme emerged as a key aspect of participants’ depressive experience. However, participants additionally provide some insight into why this occurs as highlighted by the following comment: *“For my own protection, as I said to you, I then withdraw from everybody really”* (P2, female, 65 years old).

This quote therefore highlights how some participants specifically utilise social withdrawal as a deliberate and self-protective strategy.

#### Sub-Theme 1.3: Retreating into one’s internal world (withdrawal into oneself).

In addition to attempts to completely avoid contact with the external world (global withdrawal) and reduce contact with others (social withdrawal), participants also spoke of withdrawal in the form of retreating into ‘themselves’ and their internal world in response to depressed mood: *“I’ve gone into my internal world”* (P4, male, 52 years old).

That this happens in response to a drop in mood is further clarified in the following comment: *“I just find myself withdrawing further and further inside myself when I’m feeling depressed”* (P15, female, 47 years old).

Participants spoke negatively of the sense of isolation and separation from others that this can bring, highlighting the potentially aggravating impact on depressed mood: *“Withdrawing into myself makes me feel cut off from everyone around me. Like I’m out of reach….. It upsets me and I can see it upsets my family but I can’t seem to stop it”* (P16, male, 52 years old).

### Theme 2: Perceptual detachment from one’s experience

In addition to withdrawing behaviourally as identified above, participants also describe a tendency to withdraw perceptually in response to a deterioration in mood.

#### Sub-theme 2:1: Not being aware of one’s internal experience.

Participants reported a failure to observe internal events such as thoughts, feelings and urges: *“So I don’t know…………… what I want to do or what I feel or what I think a lot of the time”* (P3, female, 57 years old).

Indecisiveness is a recognised feature of depression (17). It is possible that an inability to be aware of one’s thoughts and feelings is a contributing factor to this problem, given that knowledge of internal experiences may form part of the decision-making process: *“And whether it was a good decision to have gone or not I don’t know. I don’t know if I enjoyed it”* (P3, female, 57 years old).

Further implications of reduced awareness of internal experience are highlighted in quotes reflecting that this does not just affect distressing aspects of experience but can generalise to include all experience. The following exchange illustrates this point. Interviewer: *“What aspects of your experience get switched down?”*

*“Bad things. But actually not just bad things. Things you’re watching on the telly. Well, everything gets switched off. Like emotions, feelings, sounds”* (P4, male, 52 years old).

This example quote illustrates that reductions in awareness relate not only to internal phenomena but also to external aspects of experience as identified in the next sub-theme.

#### Sub-theme 2.2: Shutting off from external experiences.

Participant quotes describe how awareness of external experience reduces in tandem with deteriorating mood. This point is succinctly conveyed by the following observation: *“I’m less aware of external stimuli when my mood is bad”* (P6, male, 42 years old).

Changes in self-care, typically involving reduced attention to dietary intake, personal hygiene and appearance are a recognised feature of depression (17). Participants described how reduced awareness manifested itself in reduced self-care. For example, participants described missing meals, not shaving, and neglecting housework due to a failure to notice the need to undertake these tasks. This is vividly described in the following quote: *“My flat was a disgusting hovel, I just didn’t notice any of it, I didn’t clean or even see it, I didn’t care about any of that. It was the last thing on my mind. If you looked ok or your surroundings were clean, that didn’t matter. You didn’t notice whether it was raining, windy, sun, day, night whatever. I mean there could be a nuclear bomb dropped outside your window and you wouldn’t notice, you know”* (P7, female, 30 years old).

This sub-theme includes quotes relating to the reduction in awareness of external experience that accompanies depression. Participants described this as a significant and distressing aspect of their depressive experience and quotes highlight the potential role of this in maintaining depression through reducing opportunities to fully engage with pleasurable or rewarding experiences. A participant describes how this feature changes as her mood improves: *“Well, now my mood is good I notice everything. I thought it was a bit corny because I notice stupid things like the colour of the sky which I never did before. ………….. And I thought to myself how nice the sky is, and I remember thinking that recently I wouldn’t have noticed anything like that, whether it was sunny or anything. So now my mood is good I notice that kind of thing”* (P7, female, 30 years old).

### Theme 3: Intentional Reduction in Awareness

The above theme captures participants’ observations of awareness of internal and external events decreasing and increasing depending on mood state. In addition to the seemingly automatic tendency for this to happen, analysis of participant interviews highlighted that reduced awareness is deliberately sought by some as depression deepens.

#### Sub-theme 3.1: Switching off awareness to insulate oneself from painful feelings.

This sub-theme involves a deliberate constriction of awareness following onset of depression: *“If you hear and see things it’s a problem, so you don’t want to see and hear things, so you just shut yourself off from it, switch it down”* (P4, male, 52 years old).

Several activities associated with depressed mood may be regarded as behavioural strategies to escape from negative feelings, such as retreating into sleep. Others may represent efforts to directly achieve an improved emotional state, such as using alcohol or recreational drugs. However, when considered through the perspective of mindfulness, these actions were viewed not just as a way of achieving an altered state, but more specifically as deliberate strategies to inhibit awareness. For example, intentionally dulling awareness of upsetting feelings through drug use or switching awareness off altogether through sleep are tactics identified in the following quote: *“I’d self-medicate as soon as I’d get in the house. I’d be smoking a lot of weed or Valium or whatever was in house. I tried as much as possible not to feel anything, that’s why I was doing all of that……….*(P7, female, 30 years old).

*A lot of the time, an avoidance is to go to sleep so that nothing is thought of then”* (P8, female, 43 years old).

Intentionally inhabiting a state where awareness of experience is profoundly restricted is therefore described by some as a tactic for dealing with overwhelming states of stress and anxiety when mood is low. However, while reduced awareness is deliberately instigated to avoid contact with distressing phenomena, it may further reduce opportunities to engage in pleasurable and rewarding encounters. This is captured by a participant’s description of hearing a bird singing: *“Blackbird singing is a lovely sound, but when my mood is bad on the other hand, I wouldn’t hear a sound out there because I would be closing myself down. Shutting myself off in my little box”* (P18, male, 51 years old).

#### Sub-theme 3.2: Finding safety within one’s internal ‘painful’ state to avoid exposure to the external world.

While the above comments highlight participants attempts to avoid contact with painful feelings, participants also paradoxically identify that absorption in one’s distressing state can be a way of avoiding contact with external phenomena that may be considered more threatening and overwhelming. This is captured by the following quote: *“I’m more paying attention to what’s going on inside, nothing’s enjoyable, nothing fun, nothings acceptable. It’s a bit like, this is how I am and I’m staying here. I suppose it’s a bit like sulking really, I’m not intentionally doing it, but it’s like you go off and sulk for a bit. But it’s a safe place even though its hell. Yes, its torture and its hell and it’s all these bad things and yes I’d like not to have this, but at the end, its safe… but in a painful way”* (P6, male, 42 years old).

### Theme 4: Increased self-criticism

This theme contains quotes relating participants’ accounts of self-criticism and self-dislike in the context of depressed mood. Within this theme feelings of unworthiness and profound self-hatred are vividly expressed by participants and include intimations of suicidality. These views are often presented by participants as reasonable and accurate representations of their worth. Participants’ reported sense of inadequacy is partially informed by the changes in performance associated with depression, as well as their dislike of their physical appearance.

#### Sub-theme 4.1: A profound sense of worthlessness as a human being.

This sub-theme contains many similar comments with participants frequently reporting intense feelings of self-loathing and worthlessness. The profound depth of such negative feelings is powerfully expressed: *“Useless, you’re not a human being. You’re just a blob”* (P4, male, 52 years old).

The comment below succinctly conveys one participant’s total sense of failure and inadequacy and the total conviction with which she holds this opinion: *“I feel a complete failure in everything and every way. Because I am”* (P11, female, 58 years old).

That such ideas are particularly prevalent during episodes of depression is clarified in a quote from a participant who is clearly able to see the link between her mood and her self-opinion: *“I feel as if I’m not worthy of life, but that feeling goes up and down depending on how I’m feeling. When I’m very down that feeling is very prominent”* (P2, female, 65 years old).

Whilst this comment is not an explicit expression of suicidal thinking, it nevertheless appears closely related. The next comment from this participant appears to indicate a further progression towards suicidal intention, driven by profound feelings of worthlessness: *“You just think about yourself in the worst possible way, that you’re a worthless person and you don’t deserve a place on this earth and its time that you relieved yourself and everyone around you of your presence. And that’s how you feel at that lowest point.”*

Feelings of worthlessness and suicidal thinking are listed as key symptoms of depression in diagnostic manuals (e.g., 30). This is perhaps unsurprising given the potentially relentless and tormenting tone of self-critical thinking as described forcefully in the following comment: “So, its “F*** you, you’re not worth living” (P18, male, 51 years old).

#### Sub-theme 4.2: Self-blame and self-loathing for the presence of depressive symptoms.

The existence of depression and its subsequent impact on functioning in day-to-day living can additionally exacerbate self-dislike and self-criticism through the perception that this is further evidence of inadequacy and weakness. This is highlighted during a discussion about the impact of depression on one participant: *“Low self-worth and feeling useless because of all of this, and this is further evidence that I am not very… well that I’m useless”* (P6, male, 42 years old).

Another participant also describes her negative self-view arising from her inability to experience a full range of emotions: *“well life’s a mixture of all sorts of things to be experienced and, um…… responded to and made sense of……….. so, not feeling is ….. I think has rendered me sub-human”* (P3, female, 57 years old).

#### Sub-theme 4.3: Self-dislike of one’s physical appearance.

Participants spoke self-critically of their physical appearance as captured in the quote: *“This self-critical thing always walks around with me. Um, like, um, going back into the waiting room and you saw me sitting in the chair, my thoughts were “well he spoke to me on the phone and I was friendly but I bet now he’s seen me he’s disappointed, because I’m so fat, the size I am and the way I look”* (P8, female, 43 years old).

### Theme 5: Mind racing

Participants described a clear and negative change in thinking associated with depression. This theme reflects reports describing participants’ experience of relentless and overwhelming mental activity including rumination and worry that appears difficult to control. It is identified as a prominent and unwanted feature of depressive experience and its role in fuelling low mood is noted by many participants. Participants nevertheless appear compelled to engage in this activity often to the exclusion of everything else.

#### Sub-theme 5:1: Ruminating, worrying and relentless thinking.

A tendency to ruminate and worry excessively was frequently reported, with many participants vividly describing thinking becoming increasingly problem-focused, repetitious and upsetting as mood deteriorates. This point is powerfully expressed in a statement made by a participant when discussing his experience of depression: *“The idiot in my brain, also known as the gap between my ears doesn’t know better, would like to understand why the hell this is happening. I’m always trying to analyse and analyse and understand and oh my God! It’s going round and round and round and round. It drives you nuts! And the more I try to analyse it the further down I drive myself………… and the louder the voice gets “ah you complete useless piece of shit. You’re a complete waste of air. Waste of time. Go away and die!”* (P18, male, 51 years old).

This quote powerfully captures the process of ruminating and its consequences for this participant and is an experience shared by many others in this study. In addition to ruminating, an increase in worrying was also described by participants as a feature of depressed mood. A participant spoke of his tendency to worry for protracted periods of time when depressed: *“In the past I could spend weeks worrying about something when I was bad…………”* (P13, male, 41 years old).

These comments highlight the negative, repetitive, and relentless nature of thinking that can accompany an episode of depression. In addition to the change in the *type* of thinking experienced by participants, quotes also indicate that the *rate* of thinking is at times significantly increased. Participants frequently described their thoughts as becoming more rapid as noted in the following comment: *“My mind was constantly racing”* (P7, female, 30 years old).

A participant noted that this type of thinking could happen to the exclusion of other important activities: *“In the depressed state I’m extremely passive and won’t do anything like eat, or get up, or listen to the radio I just think about stuff”.* (P20, male, 57 years old).

Participants recognised the role of changes in thinking in aggravating depression as, highlighted in the comment: *“I said to my psychiatrist that the problem is the thoughts I’m having and that they’re overwhelming and they’re pushing me into more and more depression”* (P6, male, 42 years old).

#### Sub-theme 5:2: Preoccupation with thoughts preventing one from being in the present.

This sub-theme identifies how wider awareness of present moment experience is impeded by relentless ruminative and worrisome thinking. Quotes capture how attention to phenomena beyond this, including sensory experiences and events in the surrounding environment is profoundly restricted by this activity. For example, when asked about how she spent her time when last experiencing an episode of depression, one participant gave the following answer: *“Nothing could distract me from just being sad. Constantly thinking about thoughts of everything being horrible. Even if you put the telly on, I just wouldn’t watch it, yeah, I couldn’t tell you what had been on. It was just thoughts all the time”* (P7, female, 30 years old).

One participant described how preoccupation with thinking can impair performance in his job, due to an inability to attend to phenomena outside of thinking: *“That’s not very conducive to me being a responsive or entertaining presenter, because I’m too absorbed with what’s going on inside my head rather than what’s going on in the room. It’s almost like I’ve put the shutters down or something”* (P10, male, 36 years old).

Another participant expressed his view that the automatic tendency to prioritise thinking above other activities is prompted by an attempt to problem solve. He further highlighted how this leads to contact with other potentially helpful and enjoyable aspects of experience being restricted: *“I’ve looked at the television as though I’m using avoidance but after half an hour, I realise I haven’t seen a thing. Now I think, “What’s happened here?” and I’ve got no idea. The only conclusion I can come to is that my brain has again tried to solve whatever problem it’s working on at that time, rather than watching TV so I’ve missed it”* (P5, male, 66 years old).

### Theme 6: Impaired cognitive performance

A significant deterioration in cognitive performance was reported by participants who typically spoke of a slowing down of thinking, memory and concentration problems. These changes reportedly affected participants’ ability to function effectively in day-to-day life and were another factor in maintaining and aggravating low mood. Such changes also provided additional ammunition for self-criticism and feelings of failure.

#### Sub-theme 6.1: Losing the ability to find words to express oneself.

The impact of depression on memory and concentration appears to negatively effect some participants ability to express themselves as illustrated in the following quote: *“I think I’ve hit capacity, I can’t… the words don’t come to mind, that you know you’d like to use. It’s like to pick one out of the lottery, all those balls going around. If the right one pops up you think you can use that but it does seem to do with chance often. And to put it all together it sometimes doesn’t make sense”* (P5, male, 66 years old).

#### Sub-theme 6.2: Losing the ability to think.

In addition to difficulty with remembering words, this sub-theme refers to a wider problem in thinking clearly and coherently associated with depression. Participants describe difficulties organising thoughts in a structured and coherent manner: *“I seem to lose, to some extent, partly the ability to think”* (P2, female, 65 years old).

Information about this aspect of depression was elicited by asking participants about changes in intellectual functioning, but information relating to this was also forthcoming simply from asking participants about key experiences associated with depression. A participant who has been experiencing depression intermittently for many years clarifies that this change in functioning is specifically related to the onset of depression: *“Before my depression I could think more clearly than I can now. There wasn’t a problem”* (P19, male, 50 years old).

This sub-theme emerged from participants’ quotes that relate a deterioration in cognitive performance that were also reported to contribute to an increasingly negative self-perception: *“I’m a clever person but unfortunately due to depression I’m a thick bastard”* (P18, male, 51 years old).

#### Sub-theme 6:3: Reduced concentration on task at hand.

Concentration and memory problems were widely reported by participants. One participant describes below how this affects engagement in day-to-day activities: *“I need to cut a piece of wood. It’s a simple thing. You measure it, you go through the process, you get the wood you need, and you get a tape measure, a saw. You get everything there. I measure it. I measure it twice. And then I cut the wood and it’s wrong. Now first of all that’s a shock. But what it also means is that although I believe that my concentration as I’m cutting the piece of wood is right on the task in hand, I’m actually somewhere else. It’s a funny thing to say that my mind isn’t doing what I want it to do and what I’m telling it to do. It’s off, it doesn’t even let me know it’s off somewhere. It fools me. So I’m thinking I’m in the present and realise I’m not”* (P5, male, 66 years old).

Problems with memory can significantly undermine an ability to attend to the task at hand and potentially has serious and even dangerous consequences: *“I’ve not got a very good memory now. Like sometimes I’ll go to the cooker and forget that it’s hot and try to take something out without gloves on. I’ve burnt myself a few times like that. I forget what I’m doing, things that are in the cooker and that type of thing. Once I went to the cooker and I couldn’t remember how to turn it on and that…. Mad! I get confused”* (P11, female, 58 years old).

## Discussion

This study explored participants’ experience of mindfulness and how this may alter in the context of moderate-to-severe persistent depression. Thematic analysis findings suggested that participants’ capacity for a nonjudgmental, present moment awareness is severely impeded by the onset of depressed mood. Findings also highlighted how a deterioration in mood is accompanied by behavioural and perceptual withdrawal, in part initiated as a deliberate strategy to mitigate the impact of depressed mood. Further depressive experience included impaired cognitive performance, a tendency for relentless thinking and self-criticism, all of which appeared to compound and exacerbate low mood whilst driving out any premorbid capacity for mindful awareness.

To the best of the authors’ knowledge, the sample in the current study presented with the highest levels of depression recorded in studies using the FFMQ as well as with one of the lowest recorded scores on the FFMQ reported in the literature (FFMQ total: 97.4). similar scores on the FFMQ can be found in samples with ‘problematic levels of stress’ (FFMQ total: 108.91.; [40]), women with borderline personality disorder (FFMQ total: 110.36; [41]), and individuals with mild to moderate depression (FFMQ total: 113.88; 4V3). The present sample characteristics seem to suggest an inverse relationship between mindfulness and depression in individuals with moderate to severe, persistent depression.

### Mindfulness and depression: A negative relationship

The findings from the thematic analysis suggested a negative relationship between mindfulness and depression in line with previous quantitative [e.g., 42;35] and qualitative study findings [e.g., 22;23]. The themes ‘Perceptual detachment from one’s experience’ (Theme 2), ‘Intentional reduction in awareness’ (Theme 3) and ‘Preoccupation with thoughts preventing one from being in the present’ (Sub-theme 5.2) capture participants’ experience of awareness reducing in the face of deteriorating mood. Though these overarching and subordinate themes are not portraying the construct of mindfulness per se, as they lack essential mindfulness elements such as acceptance [43], they nevertheless capture key aspects of mindfulness such as a capacity for attentiveness to present moment experience [44]. Moreover, negative changes in self-acceptance and judgement captured in the theme ‘Increased Self-Criticism’ (Theme 4) reflect this central aspect of mindfulness negatively altering in the face of depressed mood.

### Mindlessness as a protective strategy

Participant extracts highlight a seemingly involuntary constriction of mindful awareness (Theme 2: ‘Perceptual detachment from one’s experience’ of both internal (Sub-theme 2.1: ‘Not being aware of one’s internal experiences’ and external experience (Sub-theme 2.2: ‘Shutting off from external experiences’ in the context of depression. However, findings captured in the theme ‘Intentional reduction in awareness’ (Theme 3) also suggest that people with persistent and substantial levels of depression deliberately initiate reductions in awareness as a strategy to protect themselves from contact with distressing phenomena. Participant comments about restricting awareness appear to relate to the capacity to observe experience including bodily sensations, sounds, smells, sights, thoughts, and emotions. Whilst the author were unable to identify literature referring to people with depressive disorder describing an intentional inhibition of mindful awareness, participants’ comments indicate that this strategy appears linked with the use of experiential avoidance (Sub-theme 3.1: ‘Switching off awareness to insulate oneself from painful feelings’ & Sub-theme 3.2: ‘Finding safety within one’s internal ‘painful’ state to avoid exposure to the external world’). Experiential avoidance consists of attempts to avoid internally generated experiences such as thoughts, feelings, and bodily sensations [45], and has been linked to a range of mental disorders [46,47; 48], including persistent depression [49].

Participants in the current study spoke of the use of drugs and alcohol as a method for reducing awareness of unwanted thoughts and feelings, as identified in the subtheme ‘Switching off awareness to insulate oneself from painful feelings’ (Sub-theme 3.1) while experiential avoidance has also been thought to be prompting the use of these as a means of escaping or distracting from aversive experiences [45]. In addition to an ‘Intentional reduction in awareness’ (Theme 3), the existence of experiential avoidance in the present study is also suggested by the theme ‘Behavioural Withdrawal’ (Theme 1) in which participants describe avoiding contact with people (Sub-theme 1.2) and external environments (Sub-themes 1.1 & 1.3) as a method of protecting themselves from negative experiences and feelings associated with depressed mood. Several previous qualitative studies have also implicated the tendency of people experiencing depression to withdraw behaviourally and socially [e.g., 50], which is likely to reduce opportunities for enjoyable activities [51] and create additional stress, further exacerbating depressed mood. Whilst withdrawal may be facilitated by a desire by the individual to avoid being seen as vulnerable or ‘weak’ [52], improving social connections and family relationships may be considered a priority goal by people with depression [53] and some participants in the current study recognised the advantages of re-engaging with social activities. However, despite recognising the potential benefits of becoming more socially and behaviourally active, participants consistently described isolating themselves from others as an immediate reaction to the onset of depression. This appears to be a practical attempt to avoid contact both physically and perceptually with unwanted experiences. While social withdrawal is a typical behavioural symptom observed in depressive disorders, such reported attempts potentially contribute to worsening mood by actively reducing opportunities for engagement in mood-enhancing activities and providing further ammunition for self-criticism and feelings of failure, as captured in the theme ‘Increased Self-Criticism’ (Theme 4).

Other experientially avoidant strategies utilised by participants in the current and prior studies include engaging in distracting activities and attempts at cognitive suppression [54]. Cognitive suppression can be conceptualised as a type of experiential avoidance, referring to the deliberate removal of thoughts from awareness [55], and is theorised to represent a strategy employed by people with a range of mental disorders, including depression, to manage the distress associated with these [56]. As such, thought suppression appears to substantially overlap with the subtheme ‘Switching off awareness to insulate oneself from painful feelings’ (Sub-theme 3.1), which reflects participants’ descriptions of an intention to avoid contact with their thoughts. Attempts to suppress thoughts have been associated with an increased risk of depression and anxiety in numerous studies [57; 58] while several study findings have demonstrated a strong positive correlation between chronic thought suppression and depression [59–62].

### Impact of rumination and relentless thinking

Thought suppression has also been positively associated with rumination in correlational analyses [63] while high suppressors have been found to experience high levels of rumination [64]. Although it seems counterintuitive that rumination and thought suppression should be positively associated, suppressing thoughts becomes particularly challenging during periods of stress and low mood, and the accompanying hypervigilance for unwanted thoughts tends to result in an increase in these thoughts [55; 58; 65]. Consequently, the paradoxical increase in unwanted thoughts resulting from failed attempts at suppression may trigger rumination in a further maladaptive attempt to effectively manage the depressive experience [64]. This is likely to be accompanied by additional self-criticism as failures in thought control may be attributed to personal weakness [66], and participants in the present study spoke of their frustration at their inability to disengage from ruminative thinking. Unwanted increases in rumination may in turn trigger efforts to cognitively suppress, creating a cycle of suppression-rumination that unwittingly aggravates depressed mood. Such a possibility would fit with existing models of depression [e.g., 67; 68] and the current study findings highlighting the simultaneous coexistence of deliberate efforts to suppress awareness and high levels of ruminative and repetitive thinking in qualitative themes, as in ‘Intentional reduction in awareness’ (Theme 3) & ‘Ruminating, worrying and relentless thinking’ (Sub-theme 5.1). The apparent dominance of ruminative and relentless thinking in response to depression [51; 69] is described by participants as a highly undesirable and upsetting aspect of depressive experience. Furthermore, absorption in agitated and relentless thinking presents another obstacle to the ability to observe present moment experience, including awareness of potentially mood-enhancing phenomena, i.e., as in ‘Preoccupation with thoughts preventing one from being in the present’ (Sub-theme 5.2), likely assisting the continuation of depressed mood.

### Self-loathing, hostility and criticism

Thematic analysis in the current study highlighted the powerful sense of self-loathing, hostility, and criticism that participants repeatedly direct towards themselves, and this is clearly a dominant feature of their depressive experience, as captured in the theme ‘Increased self-criticism’ (Theme 4). An important element of mindfulness includes the capacity to be non-self-critical and non-judgemental [43] towards unwanted thoughts and feelings (FFMQ facet Nonjudge). Given that depression is characterised by the very presence of unwanted thoughts and feelings and is usually accompanied by intense self-criticism [31], it is highly probable that the onset of depression is marked by reductions in this aspect of mindfulness, as suggested by the theme ‘Increased self-criticism’ (Theme 4) in which participants speak of a profound sense of self-loathing accompanying their depression (Sub-theme 4.1 ‘A profound sense of worthlessness as a human being’ & Sub-theme 4.3 ‘Self-dislike of one’s physical appearance’). Moreover, participants highlight that their self-criticism relates to the existence of depression as well as their perceived failure to manage this (Sub-theme 4.2: ‘Self-blame for the presence of depressive symptoms’). This sense of failure and accompanying self-criticism may prompt a drive to reduce levels of present moment awareness, to avoid contact with negative and distressing thoughts and feelings, as mood begins to deteriorate and self-critical, ruminative thinking escalates.

Participants in the present study describe a profusion of judgemental and self-critical thoughts and subsequent suppression of observation of these would appear a logical response. However, whilst deliberate suppression of awareness appears to be an attempt to manage depressive experience, it has several potential pitfalls. These are reflected in participant quotes suggesting that not only is negative material suppressed, but awareness of positive phenomena, including thoughts and feelings, is also obstructed, thereby reducing contact with experiences that may counteract depressed mood. In this way a strategy of deliberately restricting awareness to protect oneself from upsetting thoughts and feelings associated with depression appears to represent a ‘sledgehammer to crack a walnut’ approach in which this fails to be applied selectively to only negative material, potentially resulting in the exacerbation of depression rather than its intended relief. It is alternatively possible that rather than a failure to target specifically negative emotional material for suppression, participants are instead deliberately attempting to avoid all emotions, including positive ones, as has been found in prior studies of emotion suppression [70; 71]. This may in part be driven by a belief that they do not deserve to have positive experiences, a possibility supported by theme analysis in the current study (Theme 4: ‘Increased self-criticism’). The tone of self-disgust and recrimination captured in participant quotes indicates their perceived lack of self-worth and sense that they deserve to suffer in this way, as found in previous studies [e.g., 53]. Such beliefs may lead to participants seeking to suppress positive as well as negative emotions [71], an outcome that is nevertheless associated with further depressed affect [72–75].

### Changes in cognitive functioning

The present findings also revealed participants’ difficulties with organising thoughts, short term memory and aspects of cognitive functioning as captured in the theme ‘Impaired cognitive performance’ (Theme 6). Loss of concentration (Sub-theme 6.3 ‘Reduced concentration on the task at hand’), cognitive performance (Sub-theme 6.2: ‘Losing the ability to think’) and memory, including reduced fluency of speaking and ability to find words (Sub-theme 6.1: ‘Losing the ability to find words to express oneself’) are similar to sub-themes in other qualitative studies, consistent with findings in the current study [50; 51; 76]. As such, depression related declines in concentration and memory functioning combined with reduced awareness of present moment experience may negatively affect the ability to articulate experience. The ability to articulate experience is identified as another central aspect of mindfulness in the FFMQ [35], and problems with this may further contribute to low levels of mindfulness found in the current study. Participants also highlight that changes in cognitive performance may provide more ammunition for self-criticism and further aggravate depressed mood.

### Synthesis of findings

This study provided rich detail about the enduring and disabling experience of depression in people in secondary mental health services. Themes and sub-themes derived from thematic analysis indicate that people who have a protracted experience of moderate-severe depression relate several features accompanying this problem, namely, they withdraw behaviourally and perceptually, experience negative changes in thinking and become more self-critical. While many of these features are recognised as constituting diagnostic criteria for depression, when considered through the perspective of mindfulness it is apparent that participants describe a significant reduction of key elements of this form of awareness as a central characteristic of their depression. While participant reports indicate that reducing awareness can be instigated intentionally as a self-protective strategy, the resulting sense of separation from internal and external experiences is spoken of as a distressing experience and one that can take people further away from experiences that may counteract the problem. These factors potentially combine to ensure the escalation and perpetuation of depressive symptoms, rather than their amelioration.

### Study strengths and limitations

While previous qualitative studies have explored the impact of mindfulness training on participants with depressive disorder [e.g., 21], to the best of the authors’ knowledge this study appears to be the first to explore the role of mindfulness in depressive disorder from the perspective of participants who were naïve to mindfulness training. Furthermore, previous qualitative research has focused predominantly on depression in remission or in those experiencing lower levels of depression than in the current study sample. As such, this study has been able to provide an in-depth account of naturally occurring alterations in mindfulness in the context of substantial levels of persistent depression.

While the sample size was sufficient to generate the sufficient depth required for thematic analysis, and data saturation was reached, the study presents with a number of limitations. The sample consisted of a majority of male participants, whereas rates of major depressive disorder are higher in women [75]; and all participants described themselves as ‘white, British’. In light of these sample demographics, it could be argued that the sample was not representative of the wider population with this disorder. Notably, the sample was recruited as part of a clinical trial focusing on the delivery of treatments for persistent and substantial depression for those in secondary mental health services. It is possible that participants’ descriptions of level of depression were influenced by a desire to ensure eligibility for the trial and subsequent participant in the current study in order to access treatments within this. Such a possibility might result in a distorted view of depression as reported by participants.

### Future directions: Research and practice

Future studies might benefit from an exploration of changes in mindfulness during episodes of depression post mindfulness training. This would allow researchers to determine whether levels of mindfulness deliberately increased through training are sustained during subsequent episodes of depression and if this training is effectively utilised in an effort to mitigate the impact of low mood. A mixed methods design might also be utilised to index quantitatively post-intervention and relapse-related changes in mindfulness. In light of the current study findings, the quantification and measurement of related processes such as rumination, experiential avoidance, self-compassion and mindfulness may shed further light on the experience of mindfulness in depression.

Previous research identifies the potential value of offering mindfulness-based approaches to a proportion of participants with high levels of chronic and persistent depression [e.g., 18]. However, while the current study suggests that increases in mindfulness may support improved mood, thematic analysis also suggests that adopting a mindful stance towards experience is complicated by depression-related processes (e.g., rumination) impeding deployment of a non-judgemental attention towards oneself and one’s experience. Adaptations to existing mindfulness approaches for depression, such as MBCT, may improve engagement and outcomes from such programmes. For example, in light of current study findings, changes to such programmes might include shorter mindfulness sessions to account for impaired cognitive performance. Exercises and practices focusing on generating a greater capacity for self-compassion might also be incorporated to address elevated levels of self-criticism.

## Conclusions

Thematic analysis indicated that mindfulness reduces in the context of moderate to severe persistent depression. Though initiated as a self-protective strategy, participant reports suggest that decreased levels of mindfulness may maintain and intensify depressive experience. Despite growing evidence of the value of mindfulness approaches for those with more chronic and severe depression, the current study findings suggest that introducing mindfulness to those with high levels of persistent depression may be particularly challenging due to the intensity of symptomatology and persistence of depression-maintenance processes potentially obstructing access to a mindful perspective. Adaptations to existing mindfulness programmes, such as MBCT, may address some of the challenges identified by participants in this study while future research and practice would benefit from additional explorations into the impact of mindfulness training on the observed mindfulness patterns in moderate to severe persistent depression.

## Supporting information

S1 File Interview Schedule. This file includes detail of interview questions and prompts to guide semi-structured interviews with participants.
(TIF)

S2 File Example Codebook. This table includes an example of a theme and subthemes with supporting quotes.
(TIF)

## References

[1] World Health Organization. Mental health atlas 2014. Geneva, Switzerland: World Health Organization. 2015.

[2] World Health Organization. Report on mental health systems in Central America and Dominican Republic using the World Health Organization Assessment Instrument for Mental Health Systems. 2009.

[3] Lopez AD. Disease Control Priorities Project. Global burden of disease and risk factors. New York, NY: Oxford University Press. 2006.

[4] Greden J. The burden of disease for treatment-resistant depression. The Journal of Clinical Psychiatry. 2001;62(16):81–101. 11480881

[5] National Institute for Health and Clinical Excellence. Depression in adults: Treatment and management. London: National Institute of Clinical Excellence. 2022.

[6] Kabat-Zinn J. Full Catastrophe Living: How to Cope with Stress, Pain and Illness Using Mindfulness Meditation. New York: Dell Publishing. 1990.

[7] Segal ZV, Williams MG, Teasdale JD. Mindfulness-based cognitive therapy for depression: a new approach to preventing relapse. New York: Guilford Press. 2013.

[8] Teasdale JD, Segal ZV, Williams JM, Ridgeway VA, Soulsby JM, Lau MA. Prevention of relapse/recurrence in major depression by mindfulness-based cognitive therapy. J Consult Clin Psychol. 2000;68(4):615–23. doi: 10.1037//0022-006x.68.4.615 . 10965637

[9] Ma SH, Teasdale JD. Mindfulness-based cognitive therapy for depression: replication and exploration of differential relapse prevention effects. J Consult Clin Psychol. 2004;72(1):31–40. doi: 10.1037/0022-006X.72.1.31 14756612

[10] Kuyken W, Byford S, Taylor RS, Watkins E, Holden E, White K, et al. Mindfulness-based cognitive therapy to prevent relapse in recurrent depression. J Consult Clin Psychol. 2008;76(6):966–78. doi: 10.1037/a0013786 19045965

[11] Kuyken W, Warren FC, Taylor RS, Whalley B, Crane C, Bondolfi G, et al. Efficacy of Mindfulness-Based Cognitive Therapy in Prevention of Depressive Relapse. JAMA Psychiatry. 2016;73(6):565. 27119968 10.1001/jamapsychiatry.2016.0076 PMC6640038

[12] Finucane A, Mercer SW. An exploratory mixed methods study of the acceptability and effectiveness of Mindfulness-Based Cognitive Therapy for patients with active depression and anxiety in primary care. BMC Psychiatry. 2006;6:14. doi: 10.1186/1471-244X-6-14 16603060 PMC1456957

[13] Manicavasgar V, Parker G, Perich T. Mindfulness-based cognitive therapy vs cognitive behaviour therapy as a treatment for non-melancholic depression. J Affect Disord. 2011;130(1–2):138–44. doi: 10.1016/j.jad.2010.09.027 21093925

[14] Kingston T, Dooley B, Bates A, Lawlor E, Malone K. Mindfulness-based cognitive therapy for residual depressive symptoms. Psychol Psychother. 2007;80(Pt 2):193–203. doi: 10.1348/147608306X116016 17535594

[15] van Aalderen JR, Donders ART, Giommi F, Spinhoven P, Barendregt HP, Speckens AEM. The efficacy of mindfulness-based cognitive therapy in recurrent depressed patients with and without a current depressive episode: a randomized controlled trial. Psychol Med. 2012;42(5):989–1001. doi: 10.1017/S0033291711002054 22017808

[16] Kenny MA, Williams JMG. Treatment-resistant depressed patients show a good response to Mindfulness-based Cognitive Therapy. Behav Res Ther. 2007;45(3):617–25. doi: 10.1016/j.brat.2006.04.008 16797486 PMC2808477

[17] Eisendrath SJ, Delucchi K, Bitner R, Fenimore P, Smit M, McLane M. Mindfulness-based cognitive therapy for treatment-resistant depression: a pilot study. Psychother Psychosom. 2008;77(5):319–20. doi: 10.1159/000142525 18600038

[18] Barnhofer T, Crane C, Hargus E, Amarasinghe M, Winder R, Williams JMG. Mindfulness-based cognitive therapy as a treatment for chronic depression: A preliminary study. Behav Res Ther. 2009;47(5):366–73. doi: 10.1016/j.brat.2009.01.019 19249017 PMC2866254

[19] Eisendrath SJ, Gillung E, Delucchi KL, Segal ZV, Nelson JC, McInnes LA, et al. A Randomized Controlled Trial of Mindfulness-Based Cognitive Therapy for Treatment-Resistant Depression. Psychother Psychosom. 2016;85(2):99–110. doi: 10.1159/000442260 26808973 PMC4756643

[20] Mason O, Hargreaves I. A qualitative study of mindfulness-based cognitive therapy for depression. Br J Med Psychol. 2001;74(Pt 2):197–212. 11802836

[21] Langdon S, Jones FW, Hutton J, Holttum S. A Grounded-Theory Study of Mindfulness Practice Following Mindfulness-Based Cognitive Therapy. Mindfulness. 2011;2(4):270–81. doi: 10.1007/s12671-011-0070-5

[22] York M. A qualitative study into the experience of individuals involved in a mindfulness group within an acute inpatient mental health unit. J Psychiatr Ment Health Nurs. 2007;14(6):603–8. doi: 10.1111/j.1365-2850.2007.01148.x 17718735

[23] Allen M, Bromley A, Kuyken W, Sonnenberg SJ. Participants’ experiences of mindfulness-based cognitive therapy: “It changed me in just about every way possible”. Behav Cogn Psychother. 2009;37(4):413–30. doi: 10.1017/S135246580999004X 19508744

[24] Bihari JLN, Mullan EG. Relating Mindfully: A Qualitative Exploration of Changes in Relationships Through Mindfulness-Based Cognitive Therapy. Mindfulness. 2012;5(1):46–59. doi: 10.1007/s12671-012-0146-x

[25] Malpass A, Kessler D, Sharp D, Shaw A. MBCT for Patients with Respiratory Conditions Who Experience Anxiety and Depression: A Qualitative Study. Mindfulness. 2015;6(5):1181–91. doi: 10.1007/s12671-014-0370-7

[26] Malpass A, Carel H, Ridd M, Shaw A, Kessler D, Sharp D, et al. Transforming the Perceptual Situation: a Meta-ethnography of qualitative Work Reporting Patients’ Experiences of Mindfulness-Based Approaches. Mindfulness. 2011;3(1):60–75. doi: 10.1007/s12671-011-0081-2

[27] Cebolla AIT, Barrachin MTM. The effects of mindfulness-based cognitive therapy: a qualitative approach. Psychology in Spain. 2009;13(1):9–16.

[28] Williams K, Hartley S, Langer S, Manandhar-Richardson M, Sinha M, Taylor P. A systematic review and meta-ethnographic synthesis of Mindfulness-based Cognitive Therapy for people with major depression. Clin Psychol Psychother. 2022;29(5):1494–514. doi: 10.1002/cpp.2773 35912665 PMC9805101

[29] Kerr CE, Josyula K, Littenberg R. Developing an observing attitude: an analysis of meditation diaries in an MBSR clinical trial. Clin Psychol Psychother. 2011;18(1):80–93. doi: 10.1002/cpp.700 21226129 PMC3032385

[30] Tickell A, Byng R, Crane C, Gradinger F, Hayes R, Robson J, et al. Recovery from recurrent depression with mindfulness-based cognitive therapy and antidepressants: a qualitative study with illustrative case studies. BMJ Open. 2020;10(2):e033892. doi: 10.1136/bmjopen-2019-033892 32075835 PMC7044862

[31] American Psychiatric Association. Diagnostic and statistical manual of mental disorders: DSM-5. 5th ed. Arlington, Va: American Psychiatric Association. 2013.

[32] Hollyman JA, Freeling P, Paykel ES, Bhat A, Sedgwick P. Double-blind placebo-controlled trial of amitriptyline among depressed patients in general practice. J R Coll Gen Pract. 1988;38(314):393–7. 3076905 PMC1711591

[33] Hall RCW. Global Assessment of Functioning. Psychosomatics. 1995;36(3):267–75. 7638314 10.1016/S0033-3182(95)71666-8

[34] Braun V, Clarke V. Using thematic analysis in psychology. Qualitative Research in Psychology. 2006;3(2):77–101. doi: 10.1191/1478088706qp063oa

[35] Polit DF, Beck CT. Nursing Research: Principles and Methods. Philadelphia: Lippincott Williams & Wilkins. 2012.

[36] Baer RA, Smith GT, Hopkins J, Krietemeyer J, Toney L. Using self-report assessment methods to explore facets of mindfulness. Assessment. 2006;13(1):27–45. doi: 10.1177/1073191105283504 16443717

[37] Liamputtong P. Qualitative research methods. South Melbourne, Vic.: Oxford University Press. 2009.

[38] Green J, Thorogood N. Qualitative Methods for Health Research. SAGE. 2013.

[39] Use of a self-report symptom scale to detect depression in a community sample. American Journal of Psychiatry. 1980 Sep;137(9):1081–4. doi: 10.1016/S0033-3182(95)71666-8 7425160

[40] Baer RA, Carmody J, Hunsinger M. Weekly change in mindfulness and perceived stress in a mindfulness-based stress reduction program. J Clin Psychol. 2012;68(7):755–65. doi: 10.1002/jclp.21865 22623334

[41] O’Toole SK, Diddy E, Kent M. Mindfulness and Emotional Well-being in Women with Borderline Personality Disorder. Mindfulness. 2011;3(2):117–23. doi: 10.1007/s12671-011-0085-y

[42] Bohlmeijer E, ten Klooster PM, Fledderus M, Veehof M, Baer R. Psychometric properties of the five facet mindfulness questionnaire in depressed adults and development of a short form. Assessment. 2011;18(3):308–20. doi: 10.1177/1073191111408231 21586480

[43] Germer C. Mindful path to self-compassion: Freeing yourself from destructive thoughts and emotions. New York: Guilford Publications. 2009.

[44] Nyanaponika T h e r a. The Heart of Buddhist Meditation. 1962.

[45] Hayes SC, Strosahl K, Wilson KG. Acceptance and Commitment Therapy: An Experiential Approach to Behavior Change. 1999.New York: Guilford Press.

[46] Hayes SC, Strosahl K, Wilson KG, Bissett RT, Pistorello J, Toarmino D, et al. Measuring experiential avoidance: A preliminary test of a working model. Psychol Rec. 2004;54(4):553–78. doi: 10.1007/bf03395492

[47] Cribb G, Moulds ML, Carter S. Rumination and Experiential Avoidance in Depression. Behav change. 2006;23(3):165–76. doi: 10.1375/bech.23.3.165

[48] Polivy J, Herman CP. Causes of eating disorders. Annu Rev Psychol. 2002;53:187–213. doi: 10.1146/annurev.psych.53.100901.135103 11752484

[49] Barnhofer T, Brennan K, Crane C, Duggan D, Williams JMG. A comparison of vulnerability factors in patients with persistent and remitting lifetime symptom course of depression. J Affect Disord. 2014;152–154:155–61. doi: 10.1016/j.jad.2013.09.001 24183488 PMC3878770

[50] Rice NM, Grealy MA, Javaid A, Millan Serrano R. Understanding the social interaction difficulties of women with unipolar depression. Qual Health Res. 2011;21(10):1388–99. doi: 10.1177/1049732311406449 Rice NM, Grealy MA, Javaid A, 21498827

[51] Amini K, Negarandeh R, Cheraghi MA, Eftekhar M. Major depressive disorder: a qualitative study on the experiences of Iranian patients. Issues Ment Health Nurs. 2013;34(9):685–92. doi: 10.3109/01612840.2013.789942 24004363

[52] Heimpel SA, Wood JV, Marshall MA, Brown JD. Do people with low self-esteem really want to feel better? Self-esteem differences in motivation to repair negative moods. J Pers Soc Psychol. 2002;82(1):128–47. doi: 10.1037//0022-3514.82.1.128 11811630

[53] Battle CL, Uebelacker LA, Magee SR, Sutton KA, Miller IW. Potential for prenatal yoga to serve as an intervention to treat depression during pregnancy. Womens Health Issues. 2015;25(2):134–41. doi: 10.1016/j.whi.2014.12.003 25747520 PMC4393850

[54] Brownhill S, Wilhelm K, Barclay L, Schmied V. “Big build”: hidden depression in men. Aust N Z J Psychiatry. 2005;39(10):921–31. doi: 10.1080/j.1440-1614.2005.01665.x 16168020

[55] Beevers CG, Wenzlaff RM, Hayes AM, Scott WD. Depression and the ironic effects of thought suppression: Therapeutic strategies for improving mental control. Clinical Psychology: Science and Practice. 1999;6(2):133–48. doi: 10.1093/clipsy.6.2.133

[56] Wenzlaff RM. The mental control of depression: Psychological obstacles to emotional well-being. In: Wegner DM, Pennebaker JW. Handbook of mental control. Englewood Cliffs, NJ: Prentice Hall. 1993;238–57.

[57] Purdon C. Thought suppression and psychopathology. Behav Res Ther. 1999;37(11):1029–54. doi: 10.1016/s0005-7967(98)00200-9 10500319

[58] Wenzlaff RM, Wegner DM. Thought suppression. Annu Rev Psychol. 2000;51:59–91. doi: 10.1146/annurev.psych.51.1.59 10751965

[59] Spinhoven P, van der Does AJW. Thought suppression, dissociation and psychopathology. Personality and Individual Differences. 1999;27(5):877–86. doi: 10.1016/s0191-8869(99)00037-9

[60] Wegner DM, Zanakos S. Chronic thought suppression. J Pers. 1994;62(4):616–40. doi: 10.1111/j.1467-6494.1994.tb00311.x 7861307

[61] Wenzlaff RM, Rude SS, Taylor CJ, Stultz CH, Sweatt RA. Beneath the veil of thought suppression: Attentional bias and depression risk. Cognition and Emotion. 2001;15(4):435–52. doi: 10.1080/0269993004200169

[62] Wenzlaff RM, Rude SS. Cognitive vulnerability to depression: The role of thought suppression and attitude certainty. Cognition & Emotion. 2002;16(4):533–48. doi: 10.1080/02699930143000338

[63] Watkins ER, Moulds ML. Thought Control Strategies, Thought Suppression, and Rumination in Depression. International Journal of Cognitive Therapy. 2009;2(3):235–51. doi: 10.1521/ijct.2009.2.3.235

[64] Wenzlaff RM, Luxton DD. The Role of Thought Suppression in Depressive Rumination. Cognitive Therapy and Research. 2003;27(3):293–308. doi: 10.1023/a:1023966400540

[65] Wegner DM. Ironic processes of mental control. Psychol Rev. 1994;101(1):34–52. doi: 10.1037/0033-295x.101.1.34 8121959

[66] Clark DA, Purdon C. Mental Control of Unwanted Intrusive Thoughts: A Phenomenological Study of Nonclinical Individuals. International Journal of Cognitive Therapy. 2009;2(3):267–81. doi: 10.1521/ijct.2009.2.3.267

[67] Nolen-Hoeksema S. Responses to depression and their effects on the duration of depressive episodes. J Abnorm Psychol. 1991;100(4):569–82. doi: 10.1037//0021-843x.100.4.569 1757671

[68] Teasdale JD, Scott J, Moore RG, Hayhurst H, Pope M, Paykel ES. How does cognitive therapy prevent relapse in residual depression? Evidence from a controlled trial. J Consult Clin Psychol. 2001;69(3):347–57. doi: 10.1037//0022-006x.69.3.347 11495165

[69] Smith JA, Rhodes JE. Being depleted and being shaken: An interpretative phenomenological analysis of the experiential features of a first episode of depression. Psychol Psychother. 2015;88(2):197–209. doi: 10.1111/papt.12034 24974952

[70] Hayes SC, Wilson KG, Gifford EV, Follette VM, Strosahl K. Experimental avoidance and behavioral disorders: a functional dimensional approach to diagnosis and treatment. J Consult Clin Psychol. 1996;64(6):1152–68. doi: 10.1037//0022-006x.64.6.1152 8991302

[71] Beblo T, Fernando S, Klocke S, Griepenstroh J, Aschenbrenner S, Driessen M. Increased suppression of negative and positive emotions in major depression. J Affect Disord. 2012;141(2–3):474–9. doi: 10.1016/j.jad.2012.03.019 22483953

[72] Butler EA, Egloff B, Wilhelm FH, Smith NC, Erickson EA, Gross JJ. The social consequences of expressive suppression. Emotion. 2003;3(1):48–67. doi: 10.1037/1528-3542.3.1.48 12899316

[73] Campbell-Sills L, Barlow DH, Brown TA, Hofmann SG. Effects of suppression and acceptance on emotional responses of individuals with anxiety and mood disorders. Behav Res Ther. 2006;44(9):1251–63. doi: 10.1016/j.brat.2005.10.001 16300723

[74] Gross JJ, Levenson RW. Hiding feelings: the acute effects of inhibiting negative and positive emotion. J Abnorm Psychol. 1997;106(1):95–103. doi: 10.1037//0021-843x.106.1.95 9103721

[75] Nezlek JB, Kuppens P. Regulating positive and negative emotions in daily life. J Pers. 2008;76(3):561–80. doi: 10.1111/j.1467-6494.2008.00496.x 18399953

[76] Fossati P, Bastard Guillaume L, Ergis AM, Allilaire JF. Qualitative analysis of verbal fluency in depression. Psychiatry Research. 2003;117(1):17–24. 12581817 10.1016/s0165-1781(02)00300-1

