# Supplementary figures and images for: A qualitative exploration into the experience of mindfulness in moderate-severe persistent depression
Source: PLoS One. 2025 Jun 9;20(6):e0323294. doi: 10.1371/journal.pone.0323294 (PMC12148166; doi:10.1371/journal.pone.0323294)

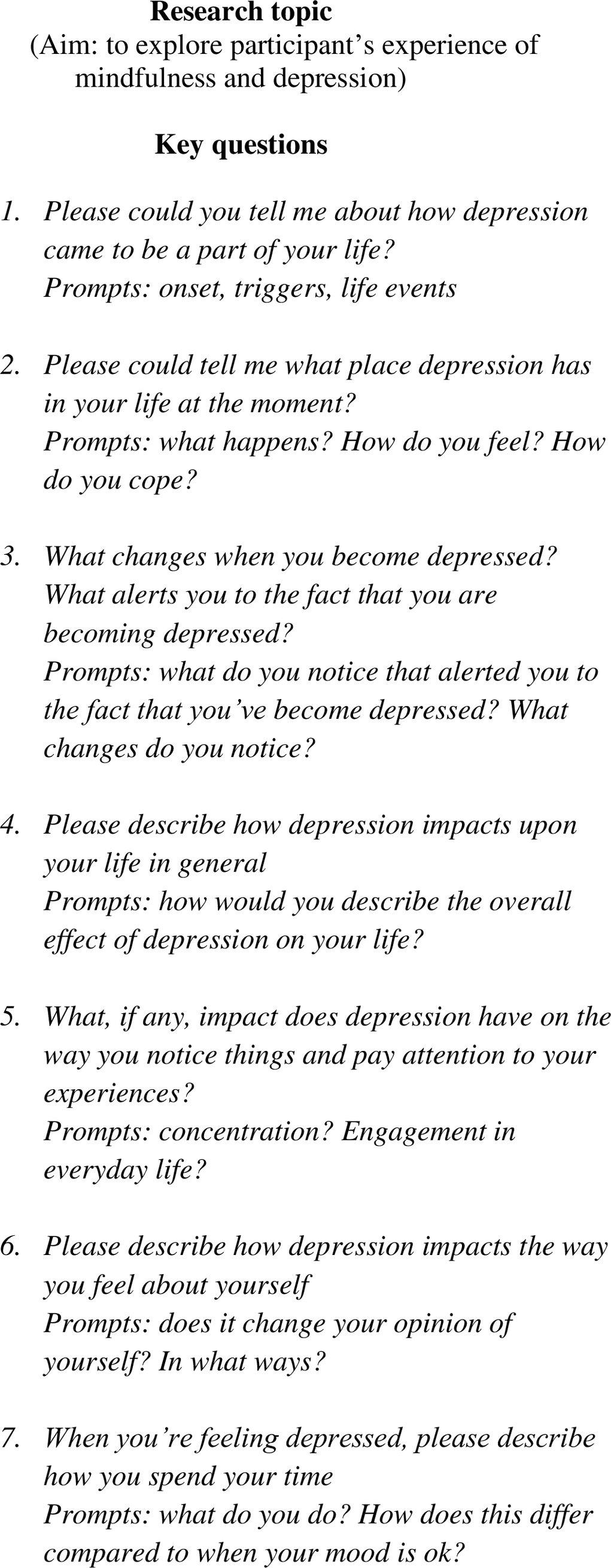

Supplement: S1 File — This file includes detail of interview questions and prompts to guide semi-structured interviews with participants. (TIF) [file pone.0323294.s001.tif]

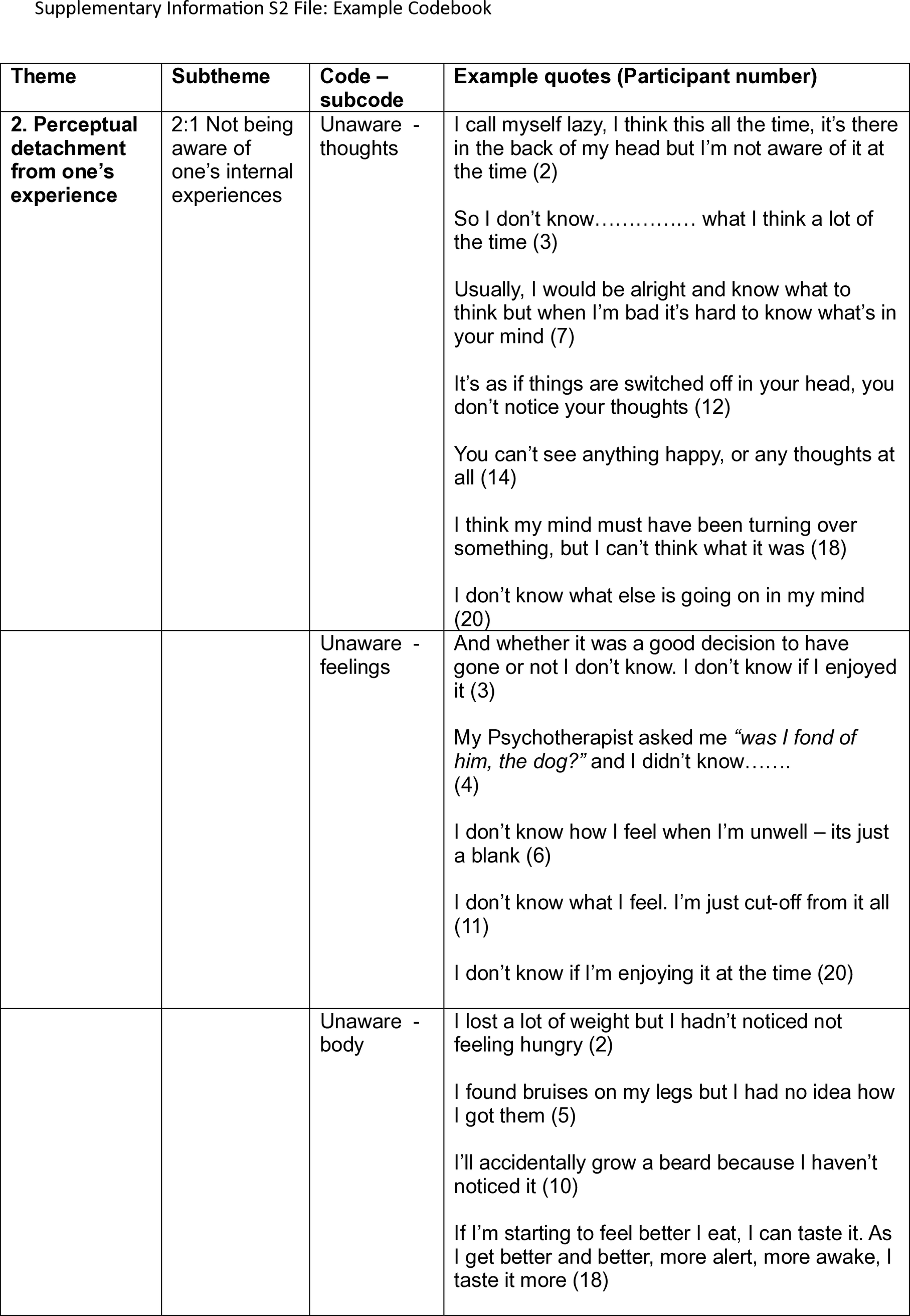

Supplement: S2 File — This table includes an example of a theme and subthemes with supporting quotes. (TIF) [file pone.0323294.s002.tif]
